# *Culex quinquefasciatus* carrying *Wolbachia* is less susceptible to entomopathogenic bacteria

**DOI:** 10.1038/s41598-020-80034-5

**Published:** 2021-01-13

**Authors:** Leonardo M. Díaz-Nieto, M. Florencia Gil, J. Nicolás Lazarte, M. Alejandra Perotti, Corina M. Berón

**Affiliations:** 1 Instituto de Investigaciones en Biodiversidad y Biotecnología (INBIOTEC - CONICET); Fundación Para Investigaciones Biológicas Aplicadas (FIBA), Vieytes 3103, Mar del Plata, Argentina; 2 Ecology and Evolutionary Biology, School of Biological Sciences, University of Reading, Reading, UK; 3 Present Address: Departamento de Biología - Instituto y Museo de Ciencias Naturales, Facultad de Ciencias Exactas, Físicas y Naturales, Universidad Nacional de San Juan, CONICET, San Juan, Argentina

**Keywords:** Microbiology, Molecular biology, Zoology

## Abstract

In an attempt to evaluate the susceptibility of the mosquito *Culex quinquefasciatus* to bacterial agents, a population naturally infected with a *Wolbachia pipientis w*PipSJ native strain was tested against the action of three bacterial mosquitocides, *Bacillus thuringiensis* subsp. *israelensis*, *Bacillus wiedmannii* biovar *thuringiensis* and *Lysinibacillus sphaericus.* Tests were carried out on mosquito larvae with and without *Wolbachia* (controls). *Cx. quinquefasciatus* naturally infected with the native *w*PipSJ strain proved to be more resistant to the pathogenic action of the three mosquitocidal bacterial strains. Additionally, *w*PipSJ was fully characterised using metagenome-assembled genomics, PCR–RFLP (PCR-Restriction Fragment Length Polymorphism) and MLST (MultiLocus Sequence Typing) analyses. This *Wolbachia* strain *w*PipSJ belongs to haplotype I, group *w*Pip-III and supergroup B, clustering with other mosquito *w*Pip strains, such as *w*Pip PEL, *w*Pip JHB, *w*Pip Mol, and *w*AlbB; showing the southernmost distribution in America. The cytoplasmic incompatibility phenotype of this strain was revealed via crosses between wildtype (*Wolbachia*^+^) and antibiotic treated mosquito populations. The results of the tests with the bacterial agents suggest that *Cx. quinquefasciatus* naturally infected with *w*PipSJ is less susceptible to the pathogenic action of mosquitocidal bacterial strains when compared with the antibiotic-treated mosquito isoline, and is more susceptible to *B. thuringiensis* subsp. *israelensis* than to the other two mosquitocidal agents.

## Introduction

The mosquito *Culex quinquefasciatus* (Diptera: Culicidae) is a known vector of pathogens of significant medical importance^1^ in Africa, Central and South America and Asia; successfully transmiting major parasites like filaria (*Wuchereria bancrofti* and *Dirofilaria immitis*), and well described viruses, West Nile virus (WNV) or St. Louis encephalitis (SLEV) and Venezuelan equine encephalitis^2–4^.

Like other mosquito species, adults of *Cx. quinquefasciatus* only fly short distances and are believed to disperse throughout the world by anthropic action. Their immature stages can be found in exposed tires that fill with water, and any type of water container on modern trucks or ships, and adults have been even detected on all kind of vehicles including airplanes or long-distance buses^5^. In Argentina, it occurs in different biogeographic regions^6^, especially urban zones^7^, being a competent vector of WNV and SLEV^8,9^.

To control mosquitoes, a number of biological methods have been applied in the past, among them the IIT (Incompatible Insect Technique) caused by *Wolbachia pipientis*. Despite its very first success on *Culex* mosquitoes in Burma, now dating over 50 years^10,11^, the *Wolbachia* IIT technique has only seen a rise in applications in the last few years. This bacterium was first detected in the common household species *Culex pipiens,* in 1924, by Hertig and Wolbach^12^. It is the most widespread bacterial endosymbiont infecting terrestrial arthropods, mainly insects, but some arachnids, freshwater crustaceans and filarial nematodes too^13^. *Wolbachia* is maternally transmitted and some of its reproductive phenotypes have been well studied, like CI or cytoplasmic incompatibility, parthenogenesis, feminization, as well as death of male offspring^14^; with CI being the most studied phenotype in mosquitoes and defined as an early form of embryonic lethality, since paternal chromosomes are lost during the first mitosis^15^.

Moreover, *Wolbachia* can counteract the effect of human pathogens within mosquito species^16^. In artificially infected *Aedes aegypti* with *w*Mel *Wolbachia* from *Drosophila melanogaster*, the mosquito ability to transmit arboviruses is reduced (dengue, chikungunya and Zika)^17,18^. *Anopheles stephensi* populations infected with *Wolbachia w*AlbB strain, from *Aedes albopictus*, are resistant to *Plasmodium falciparum*^19^ (malaria parasite), but the *Plasmodium* interference phenotype is not the same for all vectors. For example, in an *Anopheles gambiae* population transiently infected with *w*AlbB, the number of *Plasmodium berghei* oocysts was higher, increasing the risk of malaria transmission^20^. Besides, *Wolbachia*-infection of *Culex tarsalis* enhanced the infection rate of WNV^18^.

It has also been demonstrated that *Wolbachia* infection confers Diptera species some resistance to insect pathogens^21^. In experimentally infected *Cx. pipiens* mosquitoes, it is clear an upregulation of several genes for immune effector molecules involved in antimicrobial pathways^22^. However, Endersby and Hoffmann while studying sensitivity of *Ae. aegypti* to the insecticides biphentrine, temephos and s-metoprene, as well as the bio-agent *Bacillus thuringiensis*, without finding significant differences comparing *Wolbachia*^+^ and uninfected mosquitos^23^. It has also been shown that the *Wolbachia* amount is higher in *Cx. pipiens* lines resistant to organophosphorus insecticides than in susceptible mosquitoes^24^; and it has been detected that this bacterial load would be responsible for fitness effects such as preimaginal mortality, and adults of smaller body size and lower fertility^25^. The results obtained in different mosquito trials demonstrate that *Wolbachia* must be carefully assessed for use as a biological control agent. The outcomes will not only depend on the mosquito species studied but also on the *Wolbachia* strain, and these two factors would vary in their response to other chemical or biological agents applied to the control of mosquito populations.

The specific action of bacterial mosquitocidal proteins from *Bacillus thuringiensis* subsp. *israelensis* and *Lysinibacillus sphaericus* as biological insecticide has been widely researched, and not only the way of action of some of these proteins such as Cry, Bim and Cyt has been demonstrated, but also the synergistic interactions between them, increasing their toxic action or reversing the insects resistance^26–28^. It has been recently reported that the epithelial lesions produced by *B. thuringiensis* crystal proteins in *Spodoptera littoralis* could induce the reduction of the host immune response permitting the midgut bacteria replication resulting in a lethal septicemia due to the microbiota resident in the host midgut^29^. However, the interaction between the microbial flora present in mosquitoes, including symbiotic bacteria, with entomopathogenic bacteria that could modify the susceptibility to toxins produced by them or be responsible for other effects, has not yet been deeply analyzed and should be considered within the context of integrated management programs of mosquitoes.

The IIT method makes use of *Wolbachia*-induced CI, resulting in the massive release and spread of mosquito males that are *Wolbachia*-incompatible with the wild-type females^30,31^. IIT can also take benefit of the *Wolbachia*-induced CI to replace a natural population with a drive-wanted phenotype^32^. To optimize the combined application of control methods, towards a much wiser vector control, like the combination of IIT, that relies on *Wolbachia* infection, with the use of its host response to entomopathogens, more research on susceptibility of *w*^+^-mosquito lines to mosquitocidal bacteria is needed.

As a new strain of *Wolbachia* has been found naturally infecting *Cx. quinquefasciatus* in southern South America, Argentina, the aim of this study was to examine the impact of three entomopathogenic bacteria on *Cx. quinquefasciatus* larvae infected with this *Wolbachia* CI-strain. This strain is fully characterised here using metagenome-assembled genomics, PCR–RFLP (PCR-Restriction Fragment Length Polymorphism) and MLST (MultiLocus Sequence Typing) analyses; and its CI phenotype studied via crosses between wildtype and antibiotic cured *Cx. quinquefasciatus*.

## Results

### *Wolbachia *effect on *Cx. quinquefasciatus* fitness

**Figure 1:**
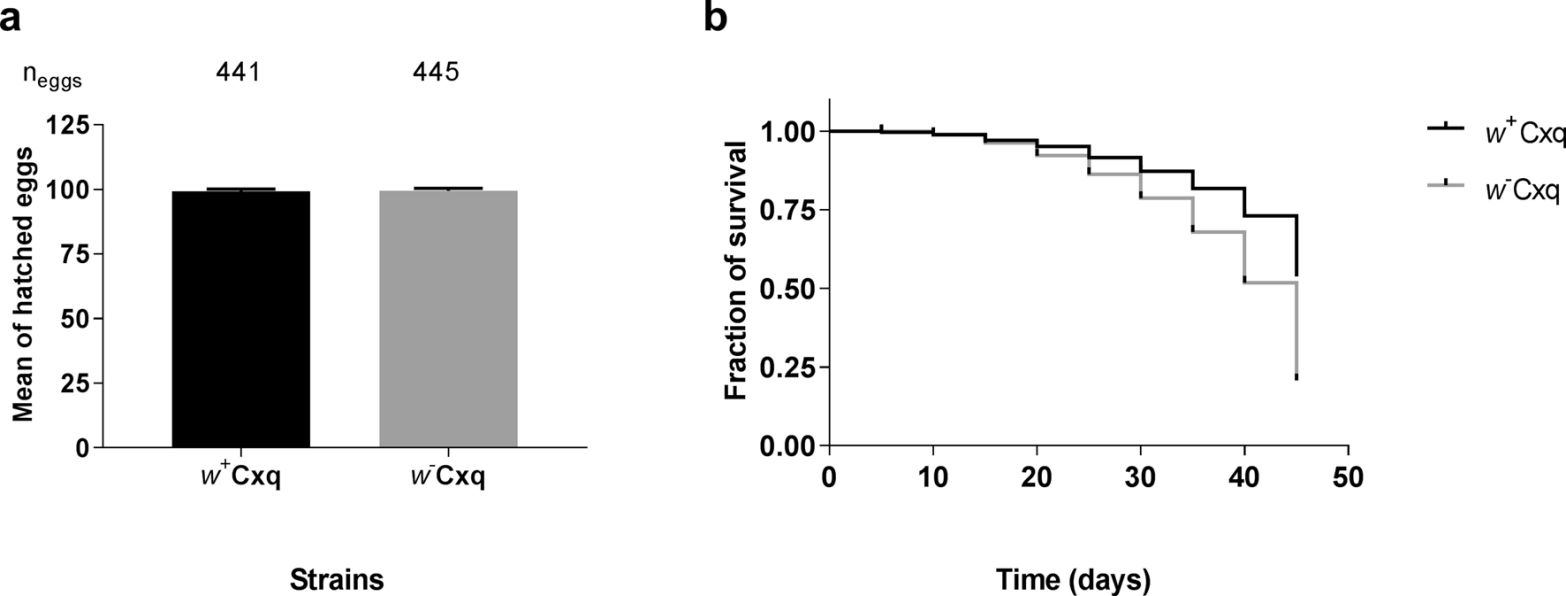
*Wolbachia* effect on fitness of *Culex quinquefasciatus*. (**a**) Mean of percentage of hatched eggs. N_eggs_: total eggs analyzed per line. (**b**) Kaplan–Meier survival curves of *Wolbachia*, (*w*^+^)- or (*w*^*−*^)-lines.

Fecundity (egg laid per female) and fertility (total eggs hatched) rates of mosquitoes infected with *Wolbachia* (*w*^+^) or treated with antibiotics (*w*^*−*^) show no significant differences for both tests (Mann–Whitney test, *p* > 0.99) (Fig. 1a). However, when analyzing time of larval development, statistical differences were observed between the mosquito lines (Fig. 1b); and Kaplan–Meier survival curves showed that the *Wolbachia* infected lines presented higher survival rates than the antibiotic-treated (Log-rank (Mantel-Cox) test: χ2 = 54.08, *p* < 0.01).

### Susceptibility of ***Wolbachia ***(***w***^+^)- or (*w*^*−*^)-***Cx. quinquefasciatus*** larvae to entomopathogenic bacteria

**Figure 2:**
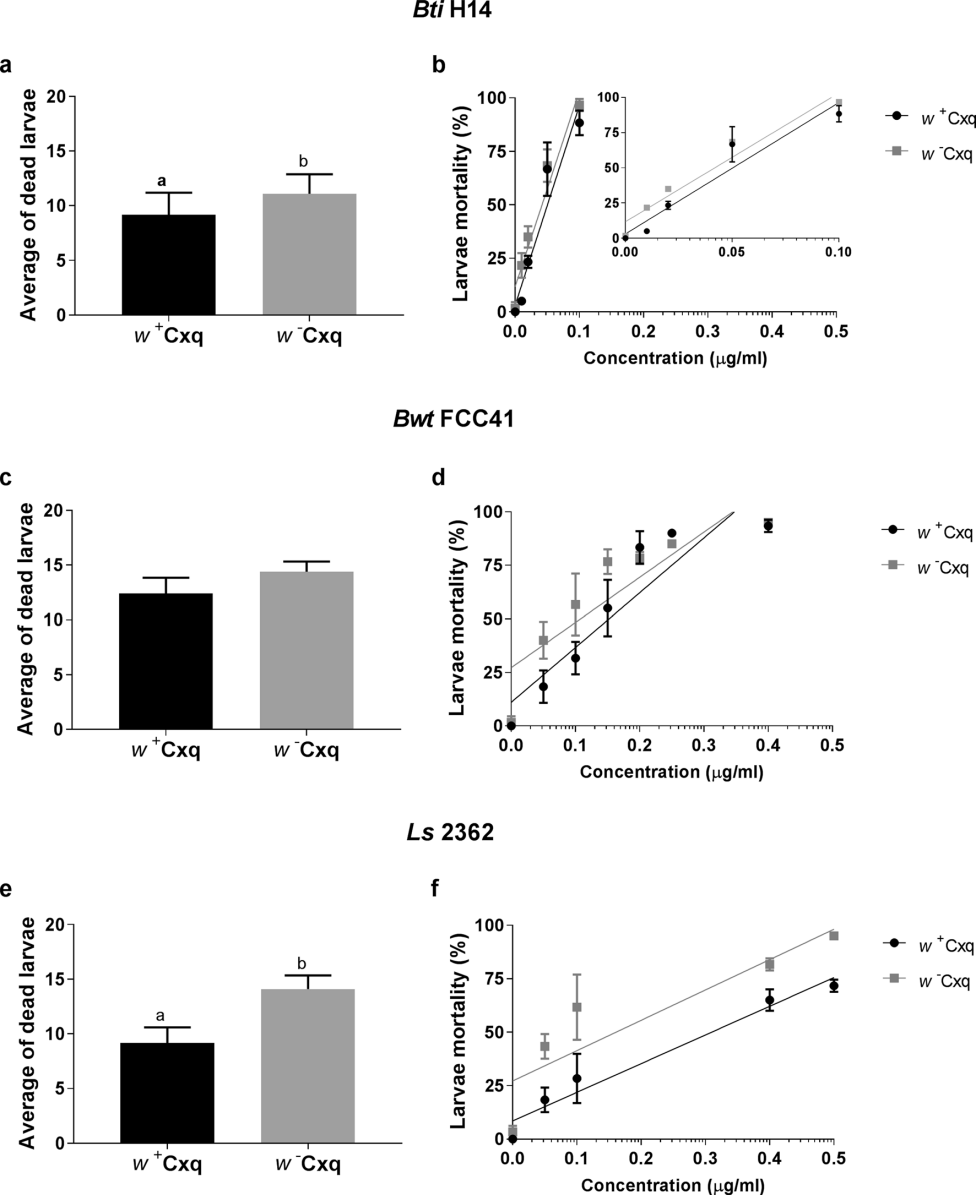
Effect of *Bacillus thuringiensis* sp. *israelensis* (*Bti* H14), *Bacillus wiedmannii* biovar *thuringiensis* (*Bwt* FCC 41) and *Lysinibacillus sphaericus* (*Ls* 2362) on (*w*^+^) or (*w*^*−*^) larvae. (**a**, **c**, **e**) Average of dead larvae (*w*^+^ and *w*^*-*^) by entomophatogenic bacteria strain. Means with different letters are significantly different. (**b**, **d**, **f**) Linear regressions of mortality per concentration between *w*^+^ and *w*^*−*^ lines with different entomopathogenic bacteria strain [Inset **b**: magnification of the curves at initial concentrations to visualize differences].

In all, the three entomopathogens used in this experiment, *B. thuringiensis* subsp. *israelensis* (*Bti* strain H14), *Bacillus wiedmannii* biovar *thuringiensis* (*Bwt* strain FCC 41) and *L. sphaericus* (*Ls* strain 2362) were required in greater concentrations for (*w*^+^)-larvae. LC_50_ values were significantly different between (*w*^+^)- and (*w*^*−*^)-larvae, and *L. sphaericus* showed the highest toxic activity difference (ratio tests, *p* < 0.05). Comparisons of LC_50_ values between lines, for each different entomopathogenic bacteria are shown in Table 1. Furthermore, mortality of *w*^+^ and *w*^*−*^ larvae were compared when they were subjected to similar concentrations of the different entomopathogenic bacteria. Mortalities were significantly higher in the antibiotic-treated lines (*Bti* H14, *p* = 0.0117; *Bwt* FCC 41, *p* = 0.0508; *Ls* 2362, *p* = 0.0001) (Fig. 2a,c,e). Plots of mortality values for each line per concentration and per strain allow visualization of differences by concentration gradient (Fig. 2b,d,f).

**Table 1:** Toxicity of *Bacillus thuringiensis* sp. *israelensis* (*Bti* H14), *Bacillus wiedmannii* biovar *thuringiensis* (*Bwt* FCC 41) and *Lysinibacillus sphaericus* (*Ls* 2362) strains against *Culex quinquefasciatus* larvae infected with *Wolbachia* (*w*^+^) or treated with antibiotics (*w*^*−*^).

Bacterial strain	*Cx. quinquefasciatus* (*w*^+^)	*Cx. quinquefasciatus* (* w*^*−*^)	Slope (± SE)	
	LC_50_ (fiducial limits)^a^	LC_50_ (fiducial limits)^a^	*w*^+^	*w*^*−*^
*Bti* H14	0.037 (0.027–0.052)	0.028 (0.025–0.031)	2.5 (± 0.2)	2.6 (± 0.2)
*Bwt* FCC41	0.128 (0.085–0.176)	0.072 (0.050–0.090)	1.7 (± 0.2)	1.8 (± 0.3)
*Ls* 2362	0.238 (0.166–0.351)	0.055 (0.035–0.075)	1.8 (± 0.2)	1.1 (± 0.2)

^a^24 hours mortality, in micrograms/milliliter.

95% confidential limits calculated by Probit statistical analysis. All values indicated were significantly different between mosquito lines according to ratio tests (*p* < 0.05).

### Molecular characterization of a native *Wolbachia w*Pip strain

According to the resulting phylogenetic tree using five concatenated MLST genes (2079 bp), the *w*Pip strain of the *Cx. quinquefasciatus* San Juan line (*w*PipSJ) of this study belongs to the B supergroup, and it has a 100% similarity to other *w*Pip strains previously reported (Fig. S1 and Table S1).

The *ank2* and *pk1* allelic profiling (for *w*Pip-I to *w*Pip-V) of the *Wolbachia* San Juan strain line resulted in a band pattern matching with *w*Pip-III group (by the specific PCR–RFLP analysis). Additionally, seven polymorphic *w*Pip genes of the *Cx. quinquefasciatus* line were analysed to allow assignment to a *w*Pip haplotype. Based on this, *w*PipSJ belongs to haplotype I (Table S2). Up to date, this is the strain showing the southernmost distribution of haplotype I and *w*Pip-III group in *Cx. quinquefasciatus* in America (Fig. S2).

**Figure 3:**
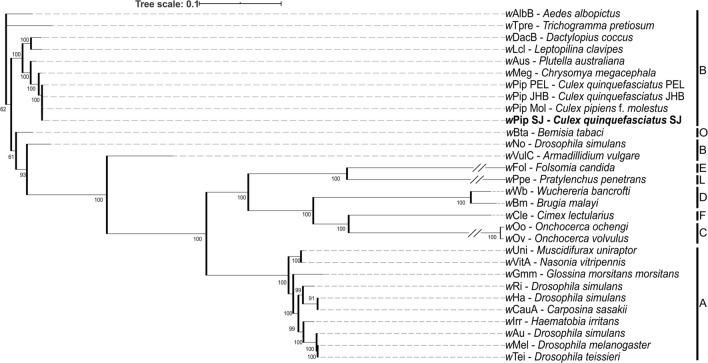
Maximum-likelihood phylogenetic tree reconstruction performed with GET_PHYLOMARKERS and IQ-TREE (500 ultrafast boostrap replicates) according to the *Wolbachia* core genome from 30 available genomes. Numbers in the branches indicate ultrafast bootstrap value, and letters on the right indicate the *Wolbachia* supergroup. The symbol // indicates a trimmed branch.

The metagenome-assembled genome of the *w*PipSJ strain consists of 1.3 Mbps, from 121 contigs and an N50 of 20,098 bp, and an average G + C content of 34.5%. This draft genome has 1171 open reading frames, 5 rRNA and 39 tRNA genes; and its total coding percentage is 82.3%. According to the core genome analysis of the *w*PipSJ and additional 29 available *Wolbachia* genomes, the phylogenetic reconstruction placed the *w*PipSJ strain in *Wolbachia* supergroup B, in the same cluster as other mosquito *w*Pip strains, such as *w*Pip PEL, *w*Pip JHB, *w*Pip Mol, and *w*AlbB (Fig. 3 and Table S3).

### Cytoplasmic incompatibility analysis of the *w*PipSJ strain

**Figure 4:**
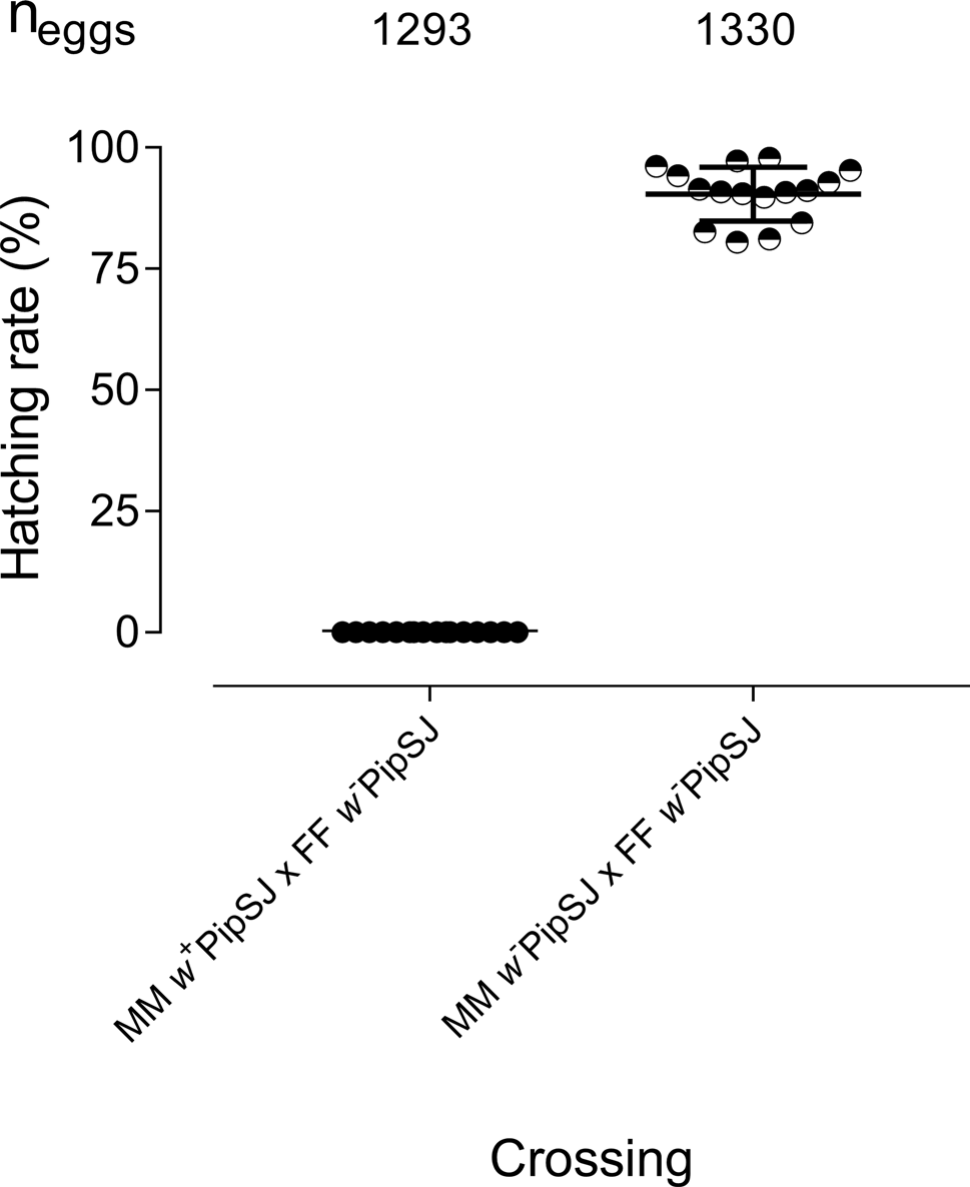
Complete unidirectional cytoplasmic incompatibility in *Culex quinquefasciatus* lines. Egg hatching percentage in crosses between the wild type (*w*^+^PipSJ) and tetracycline-treated (*w*^*−*^PipSJ) strains of *Cx. quinquefasciatus*. Sixteen couples were used in each type of crossing. Each dot represents a mosquito egg raft from a unique female, carrying approximately 150 eggs per raft. The median number of hatched eggs/egg-raft are shown in horizontal bars.

*Cx. quinquefasciatus* crosses were performed between the wild type *w*^+^PipSJ and the antibiotic-treated *w*^*−*^PipSJ populations. Crosses resulted incompatible when *w*^+^ males mated *w*^*−*^ females, obtaining 0% of eggs hatched per female, or 100% embryo mortality (Fig. 4). Both crossing types, *w*^+^FF x *w*^+^MM and *w*^+^FF x *w*^*−*^MM were compared with a t-test (unpaired), and highly significant differences were observed, *p* < 0.001. Extra confirmation of successful mating was visualised by dissection of females, showing sperm inside the spermatheca in all females with no hatched eggs (Supplementary Fig. S3). Detection of *Wolbachia* in all males from the incompatible crossings was confirmed by using PCR with the *wsp* gene primers, further description of the protocol is described in experimental procedures.

## Discussion

The *w*PipSJ strain expresses complete unidirectional CI (100% embryo mortality) in *Cx. quinquefasciatus* crossings, showing promising application for IIT strategies^30^.

The variability in the response of mosquitoes carrying the native *Wolbachia* strain and *Wolbachia*-free isoline to the different entomopathogenic bacteria, used in this work, could be explained by the differential production of putative virulence factors and extracellular degradative enzymes, such as bacteriocins, phospholipases C, enterotoxins, hemolysins, and some antibacterial proteins^27,33,34^; all this might cause modifications or imbalances in the intestinal microflora of insects that, in some way, could participate in the pathogenic activity (Table 1). Moreover, the mosquitocidal bacterial strains *Bti* H14 and *Ls* 2362 showed significant differences in the mortality of *Cx. quinquefasciatus* isoline (wild-type) naturally infected with *Wolbachia*, (Fig. 2), while there was no significant difference in the mortality of the mosquito isolines when exposed to the native or Argentinean mosquitocidal agent *Bwt* FCC41. The differences observed in the pathogenic action between bacterial pathogens could be justified with the increase in gene expression related to the mosquito immune system induced under the presence of *Wolbachia* like promoting the induction of reactive oxygen species or ROS which regulates the activation of immune genes to secrete proteins (mosquito innate response)^35^. In relation to the activation of an immune response, in a recent study on *Wolbachia* proteins, specifically WSP, it has been found that engineered WSP in *Asaia* bacteria carried by *Ae. aegypti* and *An. stephensi* mosquitoes, the mosquito (host) immune system is activated inhibiting the development of filarial parasites^36^. Another reason of the mosquito/host lower susceptibility could be related to improve nutrition in *Wolbachia* infected insects as it happens with several bloodsucking arthropods. In general, vertebrate blood as food source is deficient in some micronutrients, like B vitamins, required by hematophagous insects being supplied by the metabolism of some microorganisms associated with them as part of this microflora. For vitamins B2 and B7, complete biosynthetic pathways were found from the genome sequence analysis of *Wolbachia* while studies on diet revealed that vitamins derived from *Wolbachia* contribute to the nutrition of the host arthropod^37^.

Endersby and Hoffmann studied a number of *Ae. aegypti w*^+^ lines for their susceptibility to *Bti* H14 and, in general, only found slight differences in the response between *w*^+^ lines, but, it is not possible to know from this study how many backcrossings they received and if their genetic makeup was that of a lab naïve population^23^.

In order to demonstrate that the *Wolbachia* strain from *Cx. quinquefasciatus* in Argentina is the B supergroup described by Baldo et al.^38^, several methods were used, such as characterization of the *wsp* gene by PCR, application of MLST markers, plus, the analysis of the core genome, by comparison with other available sequences in public databases. Identification of *Wolbachia* haplotypes from different geographic regions allows to track their distribution and diversity, providing information on the evolution and dynamics of *Wolbachia* in its mosquito hosts^39^. *Wolbachia* strains vary, but the addition of a new element, a just discovered mobile putative plasmid (pWCP), made of 9.23 kbp, including 14 genes, adds even more diversity to mosquito’s *Wolbachia*, and, consequently more challenges to the dynamics of host phenotypes^40^.

For the first time, the southernmost distribution of *Wolbachia* haplotype I in *Cx. quinquefasciatus* in America is reported. This strain, *w*PipSJ, belongs to the *w*Pip-III group as it was previously described in *Cx. pipiens* in North America^41^. *Cx. quinquefasciatus* naturally infected with *w*PipSJ is less susceptible to the pathogenic action of mosquitocidal bacterial strains when compared with the antibiotic-treated mosquito isoline. The lack of fitness costs, normally associated with strong cytoplasmic incompatibility, suggests that the newly characterized *w*PipSJ from Argentina could be used in mosquito population suppression strategies based on IIT. These results encourage more research on native *Wolbachia* strains infecting mosquitoes in different parts of the world analysing the effect of them on the mosquito response to entomopathogens that could be of further help in future integrated mosquito vector management programs.

## Methods

### Permit for research with animals

Protocols for blood feeding the mosquitos on mice were reviewed and approved by the Animal Experimental Committee at the Faculty of Exact and Natural Sciences, Mar del Plata University (Institutional Committee on Care and Use of Experimental Animals (CICUAL) No. 2555-04-14). Mice were handled in strict accordance with National Health Service and Food Quality (SENASA) guidelines (Argentina) following the 2011 revised form of The Guide for the Care and Use of Laboratory Animals published by the U.S. National Institutes of Health.

### Mosquito rearing

Mosquito larvae were collected in natural breeding sites from San Juan province, Argentina. Insects were identified based on the larval fourth stage or adult females’ morphological characters and male genitalia (those reared to adult) according to Rossi et al.^42^ and Harbach^43^ respectively. Mosquito laboratory lines were established from a naturally infected population and were maintained at the Insect Biological Control Laboratory of the INBIOTEC-CONICET, FIBA (Argentina) following lab conditions such as 24 °C and 80 ± 5% RH and a 12 h light: 12 h dark photoperiodicity. Larvae were fed using fish food (Shulet Carassius). Adult stages were provided with a 10% solution of sucrose was given to adults and females were allowed to feed blood from mice.

### *Wolbachia* detection

Detection of *Wolbachia* followed PCR using the *wsp*-specific primers *wsp*-81F and *wsp*-691R^44^ from DNA extracted from single female mosquitos (twenty by colony) using PureLink Genomic DNA Mini Kit (Invitrogen, Grand Island, New York, USA) according to the manufacturer’s instructions. To verify the quality of the DNA, the amplification of the cytochrome *c* oxidase (COI) gene was used according to Folmer et al.^45^.

### Generation of antibiotic-treated *Culex quinquefasciatus* line

In order to obtain a *Wolbachia* free line, a *Cx. quinquefasciatus* wild type colony was treated with tetracycline as previously described^31,46^. A tetracycline solution (0.1 mg/mL) was used for exposure of first larvae during development. Pupae were transferred to clean water without antibiotic and reared to adulthood, and adults allowed to feed ad libitum with sucrose 10% containing 0.05 mg/mL of tetracycline hydrochloride (final concentration, Sigma, St Louis, MO; Cat. No. T33 83) for three consecutive generations. To confirm *Wolbachia* in each mosquito generation, ten individual females and ten larvae were screened using the *wsp*-specific amplification as described above. The line wild type naturally infected with *Wolbachia* (*w*^+^) and *Wolbachia* free treated with tetracycline (*w*^*−*^) were kept under same environmental conditions previously described. The antibiotic was removed in the fourth generation and the experimental work was initiated after at least three generations permitting capture of environmental microbiota, and as well as recovery from the side effects of the treatment with antibiotic. The absence of *Wolbachia* in mosquito adults cured with tetracycline was confirmed by PCR amplification of the *wsp* gene.

### Fitness parameters analysis

In order to analyze developmental differences *Cx. quinquefasciatus* lines were compared*.* Following Baton et al.^31^ 3 variables were considered to study mosquito fitness (*w*^+^ and *w*^*−*^): survival, fecundity and fertility. For that, egg laid, hatching rate, developmental stages and individual survivor number per line were daily monitored. Four rafts of eggs from each line were placed in individual containers with dechlorinated water until neonates hatched. Larvae were provided with commercial fish food (Shulet Carassius) to pupal stage. Eggs, larvae, pupae, and adults were quantified under a binocular microscope (Nikon SMZ800) until adults emerged. The full experiment was repeated 4 times considering four different generations of mosquito lines from the laboratory.

### Larvicidal activity of bacterial entomopathogens

Entomopathogenic effectivity of the *B. thuringiensis* subsp. *israelensis* (H14), *B. wiedmannii* biovar *thuringiensis* (FCC 41) and *L. sphaericus* (2362) strains was determined on larvae of *Cx. quinquefasciatus* (*w*^+^) and (*w*^*−*^) laboratory rearing lines, as previously described^47^. Twenty L2 instar larvae were placed in 20 mL of dechlorinated water. Six concentrations (0.01 to 1 µg/mL) of the spore–crystal complexes were used, and fish food was included as negative control. The bioassay was repeated three times. Examination of larvae was performed after 24 h, and these specimens were incubated at 28 °C. Using Probit analysis^48^ mean lethal concentration or LC_50_ was estimated.

### *Wolbachia* strain molecular characterization

The *w*Pip infection was characterized through the analysis of 13 different *Wolbachia* markers, which were amplified by PCR and Sanger sequenced, according to methodologies previously described. The markers were: (*i*) the *Wolbachia* surface protein gene (*wsp*)^44^; (*ii*) the analysis of five house-keeping gene sequences for the *Wolbachia* MLST methodology according to Baldo et al.^38^. For that, multiple alignment is performed with the concatenated sequences of the genes (*gatB*, *coxA*, *hcpA*, *ftsZ*, and *fbpA*, downloaded from https://pubmlst.org/wolbachia/ and detailed in Table S2). The phylogeny was estimated by Mr Bayes program, using members of all *Wolbachia* supergroups; (*iii*) the analysis of two ANK *Wolbachia* markers (*ank2* and *pk1*) by a specific PCR–RFLP assay described by Dumas et al.^41^ that allows to distinguish the 5 known *w*Pip groups (*w*Pip-I to *w*Pip-V); and (*iv*) the identification of *w*Pip haplotypes based on the polymorphism of seven genes: the former two ANK *Wolbachia* markers plus five additional genes, the DNA mismatch repair protein gene *MutL*, the ANK gene *pk2*, the methylase gene *GP12*, the putative secreted protein gene *GP15* and the regulatory protein gene *RepA*^49^. In this case, the haplotypes of each gene are identified based on the comparison of the sequence of the gene with all of the sequences of the described haplotypes methodology, according to Atyame et al.^50^.

### DNA extraction, library building construction, metagenome assembly, and analysis

Total DNA of 10 fourth stage *Cx. quinquefasciatus* larvae was extracted using the DNeasy Blood & Tissue kit (QIAGEN) and the metagenomic library was prepared using the TruSeq DNA Nano kit. Paired-end sequencing was conducted in a Novaseq platform with a 150 bp read length, generating 32 Gbp in raw reads. Quality check of reads was performed with FastQC (http://www.bioinformatics.babraham.ac.uk/projects/fastqc/). Filtering of *Wolbachia* reads was computed with Bowtie2^51^ using publicly available *Wolbachia* from *Cx. quinquefasciatus* genomes. With the extracted *Wolbachia* reads the genome was assembled using SPAdes^52^. The completeness of the genome was checked with BUSCO^53^ and an alignment with a public available *w*Pip genome was performed using Mauve software^54^ to check for genomic rearrangements and differences. The phylogenetic reconstruction was performed with GET_PHYLOMARKERS^55^ using 29 available *Wolbachia* genomes listed in NCBI genome database (Table S3). This pipeline identifies marker genes and estimates genome phylogenies from the orthologous clusters computed by GET_HOMOLOGUES^56^. The phylogeny was computed with IQ-TREE^57^ and the tree was edited with iTOL^58^.

### Crossing experiments and cytoplasmic incompatibility analysis

In order to determine the CI level, experimental crosses between FF (females) *w*^*−*^PipSJ × MM (males) *w*^+^PipSJ and FF *w*^*−*^PipSJ × MM *w*^*−*^PipSJ were performed. Pupae of each line were separated in individual glass tubes to avoid mating once adults emerged. Two days after emergence mass crosses were performed between 25 virgin adults of each sex during two days in a single cage (30 cm × 30 cm). After three days, females were blood-fed for 4 h and twenty-four hours later were removed and individually placed into smaller cages (15 × 15 cm) for oviposition. The eggs raft from each female was placed in containers with 10 mL of dechlorinated water and eggs hatched were quantified under a binocular microscope. Crossing experiments were repeated up to achieve 16 eggs raft per crossing, using five generations of laboratory mosquito lines. The spermatheca of all females that laid eggs was checked to determine the occurrence of spermatozoa and to confirm mating. Infection was detected by using the diagnostic PCR assay.

### Statistical analyses

Normality and homogeneity of variance were analyzed using the Shapiro-Wilk and Levene tests respectively^59^. The differences between % of hatched eggs per female were analyzed with Mann–Whitney U tests (*p* = 0.05). The Kaplan–Meier survival estimator was used for generating survival functions. The differences between average of dead larvae per concentration per entomopathogenic bacteria strain were analyzed with Wilcoxon matched-pairs singed rank tests. Linear regressions were plotted to observe the differences in mortality per concentration between *w*^+^PipSJ and *w*^*−*^PipSJ lines. For hatching rate data, an unpaired t-test comparison was used. Statistical analyses of the survival data were performed using GraphPad Prism version 4.01 for Windows (GraphPad Software, La Jolla California USA, www.graphpad.com). To establish the 24 h LC_50_ Log-Probit was applied, adding the 95% confidence intervals (CI) from R, specifically the package ‘ecotox’, v1.3.3, (CRAN^60^). Significant differences between the LC_50_ of the different lines were evaluated with ratio tests: if the 95% confidence interval is including 1, the difference of LC_50_ values is not considered significant^61^.

## Supplementary Information

Supplementary Information.
