# Supplementary material for: Culex quinquefasciatus carrying Wolbachia is less susceptible to entomopathogenic bacteria
Source: Sci Rep. 2021 Jan 13;11:1094. doi: 10.1038/s41598-020-80034-5 (PMC7806911; doi:10.1038/s41598-020-80034-5)
Supplement: Supplementary file 1 — Supplementary Information. [file 41598_2020_80034_MOESM1_ESM.docx]

In

***Culex quinquefasciatus* carrying *Wolbachia* is less susceptible to entomopathogenic bacteria**

Leonardo M. Díaz-Nieto^1#^, M. Florencia Gil^1^, J. Nicolás Lazarte^1^, M. Alejandra Perotti^2*^ and Corina M. Berón^1*^

^1^Instituto de Investigaciones en Biodiversidad y Biotecnología (INBIOTEC - CONICET); Fundación para Investigaciones Biológicas Aplicadas (FIBA). Vieytes 3103, Mar del Plata, Argentina. ^2^Ecology and Evolutionary Biology, School of Biological Sciences, University of Reading, Reading, United Kingdom

^#^Current address: Departamento de Biología - Instituto y Museo de Ciencias Naturales, Facultad de Ciencias Exactas, Físicas y Naturales, Universidad Nacional de San Juan. San Juan, Argentina. CONICET.

**Supplementary files**

**Table S1.** Strains ID and allele numbers from pubMLST.org used for the construction of the MLST tree.

| ***Wolbachia* strain PubMLST ID** | **Host** | ***coxA*** | ***fbpA*** | ***ftsZ*** | ***gatB*** | ***hcpA*** |
| --- | --- | --- | --- | --- | --- | --- |
| *Dmel*_A_*w*Mel | *Drosophila melanogaster* | 1 | 1 | 1 | 1 | 1 |
| *Sinv*_A | *Solenopsis invicta* | 20 | 20 | 17 | 19 | 22 |
| *Aspa*_A | *Acromis sparsa* | 2 | 26 | 21 | 23 | 26 |
| *Aape*_A_CDP21 | *Agelenopsis aperta* | 33 | 37 | 30 | 32 | 38 |
| *Iroc*_A | *Iraota rochana* | 37 | 40 | 64 | 37 | 89 |
| *Aalb*_A | *Aedes albopictus* | 2 | 3 | 10 | 3 | 2 |
| *Calt*_B | *Chelymorpha alternans* | 14 | 14 | 12 | 9 | 15 |
| *Tcon*_B_BhAvill_AK | *Tribolium confusum* | 5 | 7 | 18 | 6 | 6 |
| *Psia*_B_00189 | *Protocalliphora sialia* | 26 | 27 | 15 | 25 | 29 |
| *Aenc*_B_Ugardan | *Acraea encedon* | 11 | 12 | 11 | 9 | 12 |
| *Aepo*_B | *Acraea eponina* | 12 | 22 | 2 | 12 | 13 |
| *Cpip*_B | *Culex pipiens* | 3 | 4 | 22 | 4 | 3 |
| *Avul*_B | *Armadillium vulgare* | 13 | 13 | 9 | 13 | 14 |
| *Bmal*_D | *Brugia malayi* | 29 | 30 | 26 | 28 | 33 |
| *Clec*_F | *Cimex lectularius* | 27 | 28 | 24 | 26 | 31 |
| *Ogra*_F_100840 | *Opistophtalamus granifrons* | 31 | 33 | 48 | 30 | 35 |
| *Dtri*_F_Odo1 | *Diplacodes trivialis* | 146 | 126 | 134 | 168 | 173 |
| *Agut*_F_Odo2 | *Anax guttatus* | 146 | 126 | 134 | 168 | 174 |
| *Zang*_H | *Zootermes angusticollis* | 54 | 69 | 57 | 64 | 71 |
| *Abro*_A | *Aedes bromeliae* | 160 | 232 | 148 | 182 | 187 |
| *AfraCast1*_A | *Anastrepha fraterculus* | 1 | 1 | 3 | 1 | 1 |
| *Mafr*_B | *Mansonia africana* | 38 | 4 | 36 | 9 | 189 |
| *w*Btab | *Bermisia tabaci* | 88 | 165 | 7 | 105 | 106 |
| *w*Phar | *Petrobia harti* | 235 | 9 | 213 | 253 | 281 |
| *Gfir*_B | *Gryllus firmus* | 16 | 16 | 16 | 15 | 17 |
| *Ttai*_B | *Teleogryllus taiwanemma* | 25 | 25 | 20 | 9 | 30 |
| *Opic*_F | *Ocymyrmex picardi* | 69 | 73 | 68 | 73 | 77 |
| *Apal*_F_PanBCI | *Apoica pallens* | 63 | 78 | 61 | 65 | 73 |
| *Amet*_A | *Aedes metallicus* | 160 | 232 | 148 | 182 | 187 |
| *w*PipSJ | *Culex quinquefasciatus* SJ | 3 | 4 | 22 | 4 | 3 |

**Table S2.** Classification of the *w*PipSJ strain according to *w*Pip group and haplotype.

| **Allelic profile of two ANK *Wolbachia* markers** | | ***w*Pip Group** | **Allelic Profiles of the Seven Polymorphic *w*Pip Gene** | | | | | | | **Haplotype** |
| --- | --- | --- | --- | --- | --- | --- | --- | --- | --- | --- |
| *ank2* | *pk1* | ***w*Pip-III** | *MutL* | *ank2* | *pk1* | *pk2* | *GP12* | *GP15* | *RepA* | **I** |
| b | b |  | e | b | b | b | b | c | b |  |

**Table S3.** List of *Wolbachia* strains and accession numbers for the core genome-based phylogeny used with GET_PHYLOMARKERS.

| **Strain** | **Host** | **Nucleotide or Assembly NCBI Access number** |
| --- | --- | --- |
| *w*Mel | *Drosophila melanogaster* | AE017196 |
| *w*Ri | *Drosophila simulans* | CP001391 |
| *w*Ha | *Drosophila simulans* | CP003884 |
| *w*Au | *Drosophila simulans* | LK055284 |
| *w*No | *Drosophila simulans* | CP003883 |
| *w*Oo | *Onchocerca ochengi* | HE660029 |
| *w*Ov | *Onchocerca volvulus* | HG810405 |
| *w*Bm | *Brugia malayi* | AE017321 |
| *w*Fol | *Folsomia candida* | CP015510 |
| *w*Cle | *Cimex lectularius* | AP013028 |
| *w*Irr | *Haematobia irritans* | CP037426 |
| *w*Ppe | *Pratylenchus penetrans* | GCA_001752665.1_ASM175266v1 |
| *w*AlbB | *Aedes albopictus* | GCA_004171285.1_ASM417128v1 |
| *w*Aus | *Plutella australiana* | GCA_002318985.1_ASM231898v1 |
| *w*Uni | *Muscidifurax uniraptor* | GCA_001983635.1_ASM198363v1 |
| *w*VitA | *Nasonia vitripennis* | GCA_001983615.1_ASM198361v1 |
| *w*Pip_Pel | *Culex quinquefasciatus* Pel | GCA_000073005.1_ASM7300v1 |
| *w*Pip_JHB | *Culex quinquefasciatus* JHB | GCA_000156735.1_ASM15673v1 |
| *w*VulC | *Armadillidium vulgare* | GCA_001027565.1_ASM102756v1 |
| *w*PipMol | *Culex molestus* | CTEH01 |
| *w*Wb | *Wuchereria bancrofti* | GCA_002204235.2_ASM220423v2 |
| *w*DacB | *Dactylopius coccus* | GCA_001648015.1_ASM164801v1 |
| *w*Meg | *Chrysomya megacephala* | GCA_008245065.1_ASM824506v1 |
| *w*CauA | *Carposina sasakii* | GCA_006542295.1_ASM654229v1 |
| *w*Lcl | *Leptopilina clavipes* | GCA_006334525.1_ASM633452v1 |
| *w*Tei | *Drosophila teissieri* | GCA_005862135.1_ASM586213v1 |
| *w*Bta | *Bemisia tabaco* | GCA_003999585.1_ASM399958v1 |
| *w*Tpre | *Trichogramma pretiosum* | LKEQ01 |
| *w*Gmm | *Glossina morsitans morsitans* | AWUH01 |
| *w*PipSJ | *Culex quinquefasciatus* SJ |  |


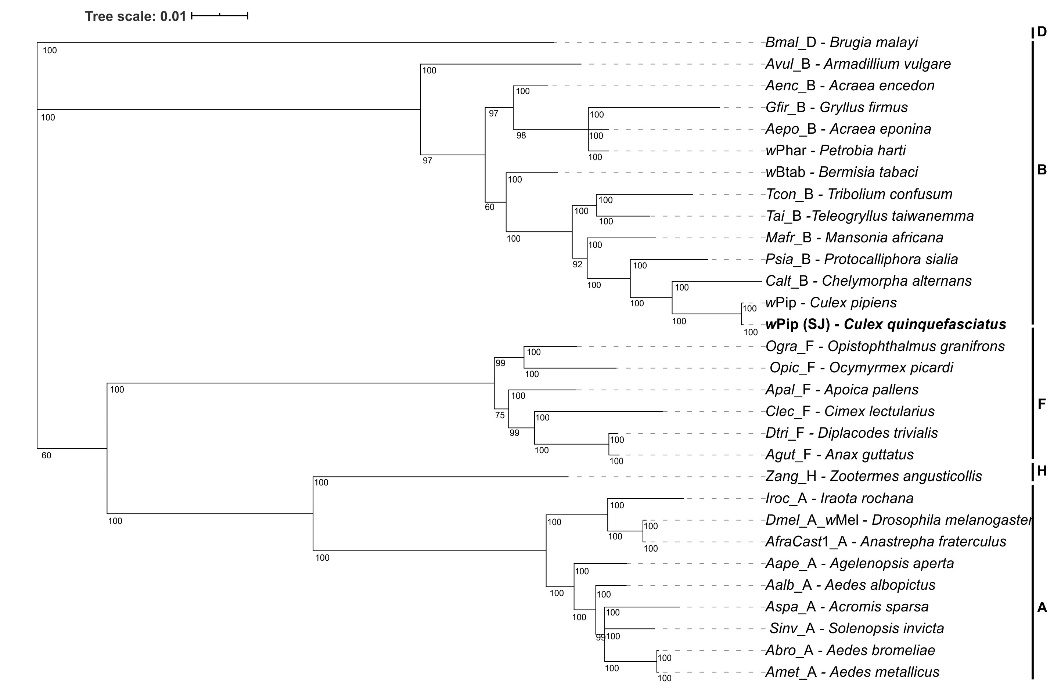


**Figure S1.** Phylogenetic tree obtained of five concatenate MLST sequences (*gatB*, *coxA*, *hcpA*, *ftsZ* and *fbpA*) by Bayesian analysis. The alignment was performed by Clustal W program including MLST sequences of all *Wolbachia* supergroups (A, B, D, F and H), while the phylogeny using Bayesian inference in Mr Bayes program. *w*Pip strain analyzed in this work (*w*PipSJ) are remarked in bold.


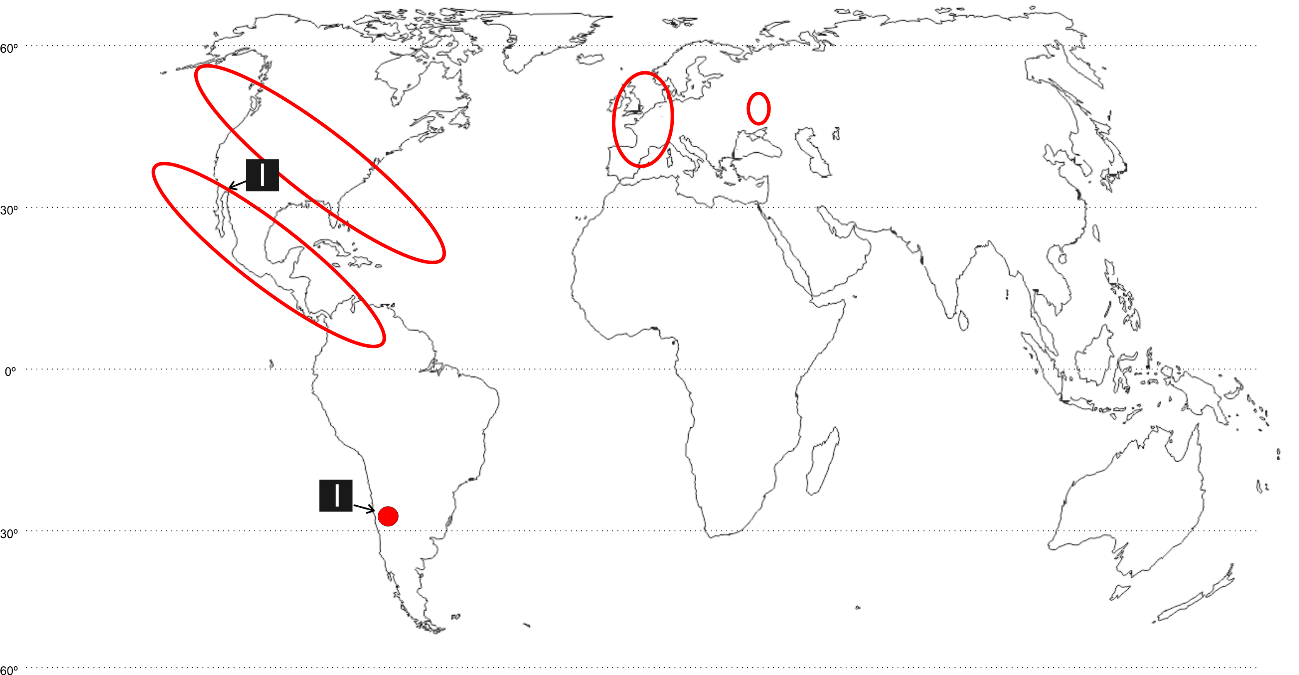


**Figure S2.** New record of distribution of *w*PipSJ strain in *Culex quinquefasciatus* from Argentina. Filled circle indicates *Wolbachia* strain present in *Cx. quinquefasciatus* line obtained from San Juan province, Argentina (this study). Empty ovals represent *w*Pip III group distribution around the world described by Atyame *et al*.^[49]^, Dumas *et al*.^[41]^ and Shaikevich and Zakharov^[62]^. Letters indicate haplotype. The map was modified from free maps of the d-maps (https://d-maps.com), using Gimp 2.10.22 program (<https://www.gimp.org/>).


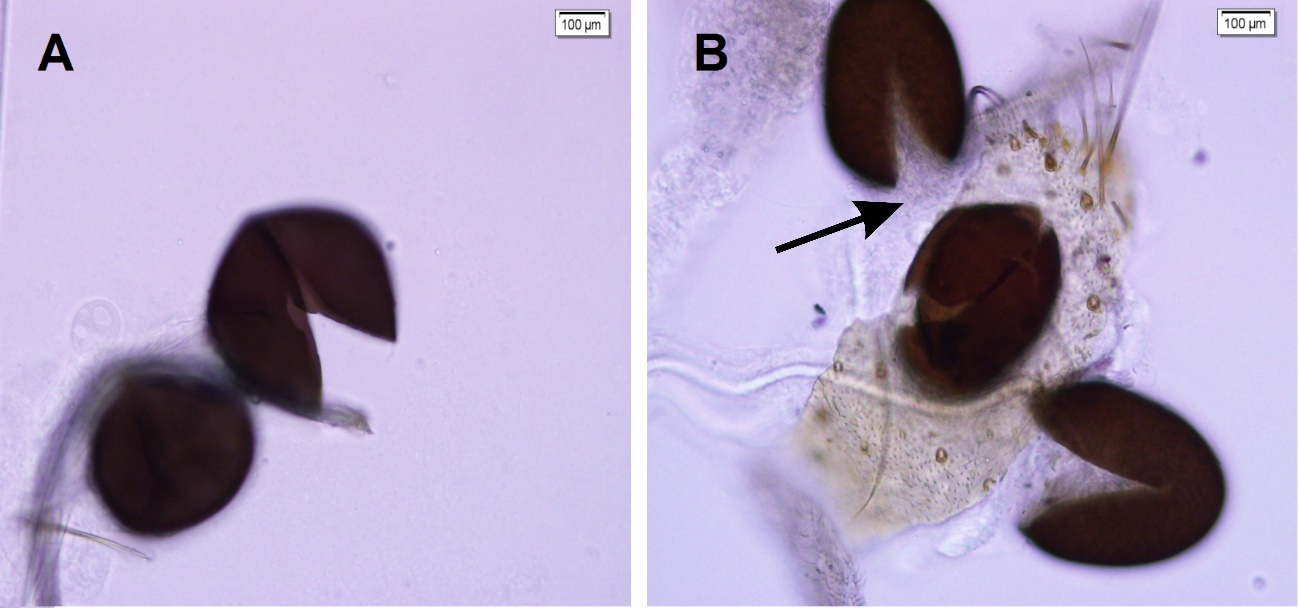


**Figure S3.** Microphotography of the spermatheca dissection of *Culex quinquefasciatus*. A. Virgin females, empty spermatheca. B. Fecundated females, spermatozoids can be seen as small filaments, indicated by black arrow.

**Suplementay References**

62. Shaikevich, E. V., & Zakharov, I. A. Coevolution of symbiotic bacteria *Wolbachia* and host mtDNA in Russian populations of the *Culex pipiens* mosquito complex. *Russ. J*. *Genet*., **50**, 1234-1237 (2014).
